# How to co-design a prototype of a clinical practice tool: a framework with practical guidance and a case study

**DOI:** 10.1136/bmjqs-2023-016196

**Published:** 2023-12-12

**Authors:** Matthew Woodward, Mary Dixon-Woods, Wendy Randall, Caroline Walker, Chloe Hughes, Sarah Blackwell, Louise Dewick, Rachna Bahl, Tim Draycott, Cathy Winter, Akbar Ansari, Alison Powell, Janet Willars, Imogen A F Brown, Annabelle Olsson, Natalie Richards, Joann Leeding, Lisa Hinton, Jenni Burt, Giulia Maistrello, Charlotte Davies, Thiscovery Authorship Group, ABC Contributor Group, Jan W van der Scheer

**Affiliations:** 1 THIS Institute (The Healthcare Improvement Studies Institute), Department of Public Health and Primary Care, University of Cambridge, Cambridge, UK; 2 The Royal College of Midwives, London, UK; 3 Royal College of Obstetricians and Gynaecologists, London, UK; 4 University Hospitals Bristol and Weston NHS Foundation Trust, Bristol, UK; 5 North Bristol NHS Trust, Westbury on Trym, UK; 6 PROMPT Maternity Foundation, Bristol, UK; 7 RAND Europe, Cambridge, UK

**Keywords:** Healthcare quality improvement, Human factors, Obstetrics and gynecology, Quality improvement methodologies, Trigger tools

## Abstract

Clinical tools for use in practice—such as medicine reconciliation charts, diagnosis support tools and track-and-trigger charts—are endemic in healthcare, but relatively little attention is given to how to optimise their design. User-centred design approaches and co-design principles offer potential for improving usability and acceptability of clinical tools, but limited practical guidance is currently available. We propose a framework (FRamework for co-dESign of Clinical practice tOols or ‘FRESCO’) offering practical guidance based on user-centred methods and co-design principles, organised in five steps: (1) establish a multidisciplinary advisory group; (2) develop initial drafts of the prototype; (3) conduct think-aloud usability evaluations; (4) test in clinical simulations; (5) generate a final prototype informed by workshops. We applied the framework in a case study to support co-design of a prototype track-and-trigger chart for detecting and responding to possible fetal deterioration during labour. This started with establishing an advisory group of 22 members with varied expertise. Two initial draft prototypes were developed—one based on a version produced by national bodies, and the other with similar content but designed using human factors principles. Think-aloud usability evaluations of these prototypes were conducted with 15 professionals, and the findings used to inform co-design of an improved draft prototype. This was tested with 52 maternity professionals from five maternity units through clinical simulations. Analysis of these simulations and six workshops were used to co-design the final prototype to the point of readiness for large-scale testing. By codifying existing methods and principles into a single framework, FRESCO supported mobilisation of the expertise and ingenuity of diverse stakeholders to co-design a prototype track-and-trigger chart in an area of pressing service need. Subject to further evaluation, the framework has potential for application beyond the area of clinical practice in which it was applied.

**Key messages**

- Much research and debate focuses on the validity and reliability of clinical tools for practice, but far less attention has been given to how to optimise their design and usability.
- We propose a framework (FRamework for co-dESign of Clinical practice tOols or ‘FRESCO’) offering practical guidance for developing prototype clinical tools, drawing on user-centred design methods and co-design principles.
- FRESCO successfully supported co-design of a prototype chart for detecting and responding to possible fetal deterioration during labour.
- By codifying existing methods and principles into a single framework, FRESCO has potential to facilitate pragmatic, flexible and inclusive co-design of clinical practice tools, but will require further evaluation.

## Background

Clinical practice tools—ranging from medicine reconciliation charts through to diagnosis support tools and track-and-trigger charts—are endemic in healthcare.^1–3^ While much research and debate focus on the validity and reliability of such tools,^4^ far less attention has been given to how to optimise their design.^5–8^ Yet, features of design, including usability,^9–11^ are among the most important influences on effective implementation.^3 7 12^ It is now clear that merely meeting technical specifications is insufficient.^6^ Critical to the effective deployment, implementation, and impact of clinical practice tools is early and continued engagement with end-users and broader stakeholders so that their priorities are addressed through design processes.^6 13^ Currently, however, thinking about how to optimise design of clinical practice tools either does not happen at all, or is deferred until far too late in the process of tool development, leading to a high level of waste associated with non-adoption or poor implementation.^14 15^ Though a range of design methods is available and widely used in other industries,^8^ their use in development of clinical practice tools has been strikingly limited.^5 6 12^ In this article, we propose that practical, action-oriented guidance could help to address this problem.

User-centred design is among the most well established of the various approaches that can support better usability of clinical tools,^16^ and is already a staple in the development of medical devices.^6 9 10 17 18^ Seeking to enhance usability of products and systems through a focus on user needs and perspectives,^16^ user-centred methods are distinguished by their systematic and typically iterative approach to optimising design through consideration of contexts of use, usability goals, user characteristics, environment, tasks, and workflow.^16 19–21^ By taking into account human capacities and limitations such as effects of stress on cognition, influence of fatigue, overload through multitasking, and limited memory capacity,^9 19 21^ user-centred approaches have potential to enable systematic consideration of safety, effectiveness, and efficiency when designing clinical practice tools.^22–24^

A user-centred approach to development of clinical practice tools is valuably complemented by co-design principles.^6 18 25^ Such principles encourage developers and users—including, for example, healthcare professionals, patients, human factors engineers, and graphic designers—to nurture collective creativity and to work in partnership.^25–27^ Application of these principles to development of clinical practice tools could strengthen or expand user-centred approaches,^6 18^ in particular by emphasising the need for early and continued engagement of end-users and broader stakeholders throughout the design process,^6 17 18 28 29^ and mobilisation of multiple forms of expertise.^6 17^ One established methodological framework for co-design describes involvement of users and developers across *pre-design*, *generative, evaluative,* and *post-design* phases (table 1).^28^ Some evidence has already demonstrated the usefulness of this approach to developing products and systems for healthcare.^29 30^

**Table 1:** The methodological framework for co-design by Sanders and Stappers^28^

Co-design phase	Explanation
Pre-design	Seeking feedback from users about their experience of using products and systems, and sensitising those involved to the problem to be addressed in the design process
Generative	Testing and refining ideas, insights and concepts with users, so that probes or prototypes can then be developed and explored for their technical and social feasibility
Evaluative	Assessing with users, in formative or summative testing, the effects and effectiveness of the developed prototypes
Post-design	Examine how users experience the design when using it in practice, with a view to evolve it in a future design cycle based on identified needs, habits, and use patterns

One example of a pressing need for improving usability and design processes is found in track-and-trigger charts for detecting and responding to patient deterioration.^12 31 32^ These widely used charts are based on the principle that there may be periods during which clinical deterioration is detectable by ‘tracking’ a predefined set of clinical parameters over time, with specific thresholds ‘triggering’ action.^33^ Track-and-trigger charts are particularly likely to benefit from user-centred design, since they seek to support clinical decision-making and action in often pressurised situations where clarity around responding to a potentially deteriorating situation is essential, and where human capacities and limitations (eg, memory capacity, effects of stress on cognition) are key influences on patient safety.^12 34^ Despite their potential value, track-and-trigger charts have been challenged by issues in acceptability, adoption, and use.^35–37^ These issues are likely to be linked to suboptimal design,^12 34^ including inadequate user involvement prior to implementation.^32 37 38^

Despite burgeoning literature on both user-centred design (and variants, including human-centred design) and co-design,^17 18 22–24 26–30 39^ practical guidance for combined use of these methods and principles in the development of clinical practice tools remains limited. In this article, we address this gap. We propose a five-step framework with recommended actions for each step, and we describe a case study of its application in developing a prototype track-and-trigger chart.

## Methods

The FRamework for co-dESign of Clinical practice tOols (FRESCO) we propose seeks to codify existing user-centred design methods and co-design principles into a single framework to guide the development of clinical practice tools to the point of readiness for large-scale testing (table 2). FRESCO recognises that development of tools usually benefits from iterative prototyping. Accordingly, it includes user-centred methods for formal prototype testing,^11 13 19 40^ and application of the co-design principle of using prototypes as tools for discovery, understanding, and learning.^28 41 42^

**Table 2:** The FRamework for co-dESign of Clinical tOols

Step	Co-design phase and user-centred method	Level of involvement	Actions
1. Establish a multidisciplinary advisory group	Pre-design phase of co-design^28^	Design partner^18^ as part of advisory group	Start with involving and preparing users and other stakeholders involved in the co-design process,^30^ by: Establishing a diverse group that represents the various forms of knowledge, including lived expertise and lived experience, needed to co-design the tool (eg, healthcare professionals, patients, human factors engineers, graphic designers).^6 7 13^ Ensuring sufficiently diverse and representative membership through use of purposeful and creative outreach, engagement with networks, inclusive methods of recruitment, and attentiveness to addressing potential power imbalances.^43 44^ Nurturing collective creativity from the start of the process through inclusive approaches and high-quality facilitation.^47 48^ Allocating roles and responsibilities across different stages of work to support efficient and effective decision-making.^45 46^
2. Develop initial drafts of the prototype	Pre-design and generative phase of co-design, including concept prototype development^28 41^ User-centred method of exploring context of use^19–21^	Informant^18^ as part of advisory group	Seek to understand stakeholders' experiences of the work system under consideration,^19–21 49^ by: Using formative usability evaluation,^11 19^ such as heuristic evaluation by a usability specialist,^11 34^ to determine which aspects of a design are likely to work well or not. Developing at least two initial draft prototypes of the tool based on usability heuristics^11 34^ and analysis of the context of use of the tool informed by experiences of users.^20 49^ Employing design elements that help users explore differences between the prototypes, including dimensions such as data density, graphical vs text-based notation, and extent of colour coding.^54 55 59^
3. Conduct think-aloud usability evaluations	Prototyping during the *generative* phase of co-design^13 28 41^ User-centred method of think-aloud evaluations^11 19^	Design partner^18^ as part of advisory group Tester^18^ as participant in think-aloud evaluations	Aim to work with representative end-users to understand processes of cognition, identify usability difficulties with their design-based causes and improve draft prototypes, by: Recruiting a sample representative of people who would be using the tool in clinical practice.^6 11 19^ Conducting recorded formative design usability evaluations using think-aloud techniques and a clinical scenario, to enable participants to verbalise their thoughts and experiences when using the initial draft prototypes.^10 11 19^ Examining the balance between sufficient information for task completion and preventing mental overload caused by too high data density,^54^ through eliciting participant preferences and reasons for wanting to include or exclude information. Interviewing the participants after the think-aloud session to generate further ideas to improve the draft prototypes.^6 10 29 56^ Analysing the think-aloud exercise and interviews.^56–58^ Using the analysis and advisory group discussion to generate an improved draft prototype.^13 41 42 46^
4. Test the prototype in clinical simulations	Prototyping during the *evaluative* phase of co-design^28 41^ User-centred method of simulation^7 40 60 61^	Design partner^18^ as part of advisory group Tester^18^ as participant in clinical simulations	Test the tool in approximations of real-life settings to enable safety checks, understand how the tool might be used in practice, and identify how to improve work systems, by: Creating a realistic clinical activity based on a simulation framework,^63 84^ including taking advantage of using simulation to reproduce rare but potentially catastrophic events or conditions.^62^ Selecting diverse healthcare settings, and inviting relevant healthcare professionals and service users (as actors) to take part in the simulations.^7 60 61^ Facilitating and recording the simulation, ideally running the same clinical scenario twice: once with a team providing usual care, and once with a different team using the draft prototype,^60 64^ while conducting ethnographic observations.^10 65^ Debriefing clinically with the teams and service users,^7 66 67^ followed by focus group discussion to generate further ideas to improve the draft prototype.^6 10^ Analysing the recorded simulations and focus group discussions.^19 57 58 70 71^ Using the analysis and advisory group discussion to generate a near-final draft prototype.^13 41 42 46^
5. Generate a final prototype informed by workshops	Evaluative phase of co-design^28^	Design partner^18^ as part of advisory group	Resolve remaining issues with involvement of representatives of all relevant stakeholder groups,^6 13 42^ by: Conducting one or more facilitated workshops with relevant stakeholders (eg, advisory group members) to obtain a final round of feedback.^10 13 17 18 39 41 46^ Using facilitators to support agenda-setting, procedures and consensus rules,^72 73^ and remaining mindful of power dynamics.^44^ Agreeing and finalising the prototype with the advisory group.^13 41^

Using five steps outlined in table 2, FRESCO aims to facilitate a process of collective creativity through structured co-design activities,^17 18 29 39^ with involvement of users, developers, and other stakeholders in roles of design partners, informants, or testers.^18^ This process is informed throughout by findings from systematic user-centred evaluations (see steps 2–4 in table 2).

The first step is to establish a multidisciplinary advisory group that offers voice to a diversity of experience and expertise throughout the process (see step 1). Following a *pre-design* phase of co-design (see steps 1 and 2), FRESCO facilitates proceeding through a *generative* phase (including gathering ideas from users based on concept prototypes produced by developers, see steps 2 and 3) to an *evaluative* co-design phase (including testing of co-designed prototypes, see steps 4 and 5). The movements from pre-design to generative to evaluative co-design phases align with, and are informed by, key user-centred design techniques such as heuristic evaluation (see step 2), think-aloud usability evaluations (see step 3) and simulations (see step 4). The last step of FRESCO aligns with completing the *evaluative* phase of co-design (see step 5), working towards a final prototype ready for further testing in real-life settings as part of the *post-design* phase (see table 1).

We used FRESCO in a case study, aiming to co-design a track-and-trigger chart for detection and response to suspected intrapartum fetal deterioration (Box 1).

Box 1 **Case study: Avoiding Brain Injury in Childbirth (ABC) programme**

In 2021, the UK’s Department of Health and Social Care commissioned the Avoiding Brain Injury in Childbirth (ABC) programme, a collaboration between the Royal College of Midwives (RCM), Royal College of Obstetricians and Gynaecologists (RCOG), and The Healthcare Improvement Studies Institute at the University of Cambridge. Colleagues from these institutions formed the ABC programme team.

A key aim of the ABC Programme was to co-design a standardised approach for detecting and responding to possible fetal deterioration during labour, including a track-and-trigger chart. The need for this work had been identified as critical and urgent because problems in intrapartum monitoring and response remain major and persistent hazards in maternity care, contributing to poor outcomes at birth and clinical negligence claims.

Current approaches to fetal monitoring during labour focus primarily on assessment of fetal heart rate features, which can be done either using intermittent auscultation (for lower-risk labours) or electronic fetal monitoring with cardiotocography (for higher-risk labours). A key innovation of the ABC programme was to combine monitoring of fetal heart rate features with other evidence-based intrapartum risk factors into a track-and-trigger tool, informed by earlier work of a task force of the RCM and RCOG. The intention of the ABC programme was to co-design an improved prototype tool, ready for deployment in a future national programme of testing, implementation, and evaluation.

## Results

**Figure 1:**
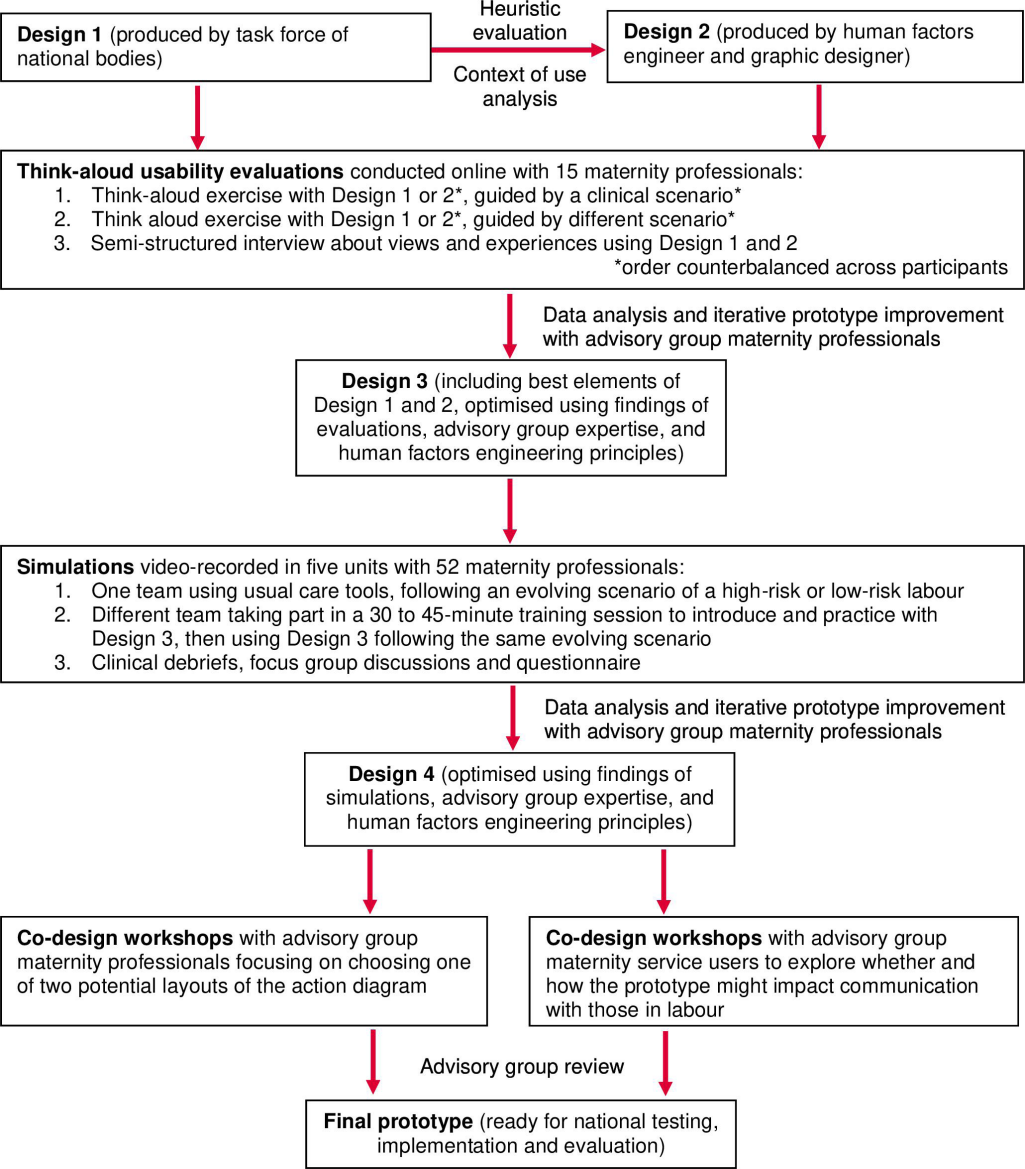


Below, we explain how each step of the framework guided the Avoiding Brain Injury in Childbirth (ABC) programme’s co-design of a prototype chart for detecting and responding to suspected fetal deterioration during labour (figure 1).

### Step 1: establish a multidisciplinary advisory group

Optimising a tool for detecting and responding to possible fetal deterioration during labour requires access to a range of expertise and experience, including scientific knowledge, clinical expertise, lived experience of labour and using maternity services, graphic design, human factors/ergonomics, and social science. We identified individuals with one or more of these forms of expertise or experience using intentional outreach and inclusive methods of recruitment.^43^ We sought to be purposeful in ensuring diversity as well as addressing the potential for power imbalances.^43 44^ For example, we included a mix of seniority among the maternity professionals and ensured that service user representation included multiple viewpoints. As detailed in online supplemental file 1, the group included the following:

10.1136/bmjqs-2023-016196.supp1 Supplementary data

- twelve maternity professionals (six midwives and six obstetricians),
- five maternity service users (representing a range of maternity experiences and experience of advocating for improvement and inclusion of under-represented voices), and
- five other specialists (human factors engineer with expertise in user-centred design, graphic designer, consensus-building specialist, and two specialists in facilitating patient and public involvement [PPI]).

As part of the *pre-design* phase (including preparation of the group for the co-design process),^28^ roles and responsibilities across different stages of work were explicitly allocated to support efficient and effective decision-making.^45 46^ This, for example, meant that not all advisory group members needed to be involved in all activities of all steps, as further detailed in figure 1 and across steps 2–5 below.

The group’s specialists in human factors engineering, consensus-building, and PPI facilitated or led exchanges, meetings, workshops, and other co-design activities.^47 48^ The PPI facilitators were particularly important in ensuring that everyone’s voices could be heard during meetings,^43 44^ as well as facilitating separate activities for maternity service users, in the interests of addressing potential power imbalances. The activities of the advisory group that were part of the *generative* and *evaluative* co-design phases^28^ are further detailed across steps 2–5 below.

### Step 2: develop initial drafts of the prototype

In accordance with the *pre-design* and *generative* phase of co-design,^28 41^ we set out to understand stakeholders’ experiences of the work system under consideration.^19–21 49^ This included developing two alternative prototypes of the track-and-trigger tool to explore which design elements worked best for maternity professionals.^13 49^

Prior to the programme, a task force from two national bodies (Box 1) had developed an initial draft prototype (‘Design 1’, see figure 1 and online supplemental file 2). It included the evidence-based clinical information required for detection and responding to possible fetal deterioration, but was focused more on clinical content than on design. A human factors engineer evaluated the draft prototype against usability heuristics,^34 50 51^ while alert to the contexts of use^20^ such as intended or expected users, tasks, physical environment, social and organisational milieu, and technical and environmental constraints.^19–21^

A second prototype (‘Design 2’, see figure 1 and online supplemental file 2) was then developed with support of the graphic designer, based on the clinical information and heuristic evaluation of Design 1 as well as the factors identified in analysis of the context of use description of the tool (see overview in online supplemental file 3).^20^ Design 2 applied established user-interface design principles (detailed in online supplemental file 3), such as the need to be attentive to limitations of memory and attention while executing a task,^51–53^ and the need for consistent use of colour coding and for grouping-related information together.^34 50–54^

Though the clinical content of both designs was the same, they used alternative page formats, colours, font types and sizes, ways of recording observations, visuals indicating actions and information structures (see details in online supplemental file 3). These alternatives were designed to enable comparison and to prompt discussions with participants about preferences in subsequent think-aloud usability evaluation (see step 3 below).^55^

### Step 3: conduct think-aloud usability evaluations

As part of the *generative* co-design phase,^13 28 41^ we conducted think-aloud formative usability sessions with 15 maternity professionals of varied backgrounds (online supplemental file 4).^10 11 19^ Asking people to work with designs 1 and 2 (see figure 1), the sessions were aimed at understanding processes of cognition and identifying usability flaws (and their causes) with a small group of representative end-users.^11 19 46^

In advance of the session, participants received print-outs of the two draft prototypes, examples of the drafts with recorded observations, and clinical scenarios. Each session took part during a video call hosted on an online platform^1^, and was facilitated by a moderator (trained interviewer or human factors engineer). Sessions were organised so that designs 1 and 2 were covered in a sequence counterbalanced across participants to mitigate order effects. The moderator started with an exercise to encourage the participant to think aloud in describing their experiences while interacting with the prototype,^10 11 19^ with prompts used to elicit experiences of particular design elements. Following this think-aloud exercise, the moderator used a semistructured interview guide (see online supplemental file 4) to ask about preferences for elements of one of the designs, elicit further views on design aspects that could be improved and use of the chart in practice.^6 10 29 56^

The sessions took about one hour each. They were audio-recorded and transcribed verbatim. Analysis focused on preferences for elements of the two designs and identification of design principles to guide future prototype iterations.^56–58^ Participants preferred the detail contained within Design 1, but found Design 2 easier to complete and interpret (table 3). These findings reflected the tension between high data density and information overload.^54^ They highlight that a particular consideration in developing clinical practical tools is striking a balance between: (1) including sufficient information to support task completion, and (2) preventing high data density that can increase search times and mental workload, particularly if information is poorly structured.^59^ Further qualitative analysis identified five requirements to inform further prototype iterations and future implementation (table 4), such as the need for optimising flow of information.^54^

**Table 3:** Examples of analysis on the number of participants who preferred specific design elements of Design 1 versus Design 2 (see online supplemental file 1 for details on design elements)

Design element	Illustrative quote/s	Content analysis
Tabulated text with YES/NO (Design 1) vs coloured row format with dots and lines (Design 2)	‘I think Tool 2 would be better, because it is easier, it’s just drawing lines […] Tool 1, you actually have to write, yes, no, all the, obviously, figures, so it takes a bit longer, I think.’ (Midwife) ‘When there’s a peak in the line, it’s easy to see where there is a problem.’ (Obstetrician)	12 out of 12 participants who commented on this element preferred Design 2
Six timeslot columns on one chart (Design 1) vs 16 timeslot columns on one chart (Design 2)	’What have you got, six hours on the first one, you’ve got more haven’t you on this second one. That’s an advantage on the second one for sure because six hours is quite limiting, isn’t it, not many people have a baby in six hours, particularly if they’re high risk and on the CTG.’ (Midwife)	11 out of 11 participants who commented on this element preferred Design 2
Flow chart action diagram (design 1) vs actions described in boxes adjacent to recordings (Design 2)	’I’m a little bit confused on this form [Design 2] as to what…the other form, the flow chart made it a little bit easier what to do.’ (Midwife)	7 out of 11 participants who commented on this element preferred Design 1
Rows with detailed fetal heart rate features (Design 1) vs rows combining related features such as decelerations and variability (Design 2)	‘[…] the degree to which you’ve got different concerns at different levels would mean that you were less or more concerned about the CTG. So I think this [Design 2] really oversimplifies the CTG too far.’ (Obstetrician)	9 out of 11 participants who commented on this element preferred Design 1
Inclusion of ‘start of labour risk assessment’ on the chart (Design 1) vs not presenting this assessment on the chart (Design 2)	’You're not going to put all the previous pregnancy bits in a risk assessment for this pregnancy. Certainly significant medical history might be useful. Has she got foetal growth restriction because she’s got a medical problem? Or is it a pregnancy related problem?’ (Obstetrician)	9 out of 9 participants who commented on this design element preferred Design 1
Aesthetics such as colours and font size of Design 1 vs Design 2	‘This one [Design 1] looks slightly more anxiety inducing. It is very busy.’ (Midwife) ’The whole look of it and feel of it [Design 2] feels more simple, it’s more relaxing.’ (Midwife) ’The orange colour [used in Design 2] you know that obviously there is a problem. So you need action.’ (Midwife) ’I think it’s all clearer [in Design 2], a bit bigger. Yeah, I have no concerns about the font and the clarity of tool B [Design 2]. I suppose when you’re just glancing at it overall, it’s really easy to see where the issues were.’ (Obstetrician)	13 out of 13 participants who commented on this design element preferred Design 2

CTG, cardiotocography.

**Table 4:** Identified requirements to inform further prototype iterations based on qualitative analysis of the think-aloud exercises and follow-up interviews

Requirement	Summary of supporting data
The chart must minimise duplication of effort	Participants stressed how any new tool and system must reduce rather than increase burden on maternity professionals, including fitting it with existing required intrapartum documentation.
The chart must contain clearly defined parameters	Participants identified potential for confusion and variation as a result of poorly defined or operationalised clinical risk factors.
The content and layout of the chart must reflect workflow	Participants expressed a diversity of opinions on which ordering of observed risk factors would be the easiest to complete within clinical practice. The prototype would benefit from further understanding the optimal ‘flow’ of information on the charts.
The chart must include robust escalation processes	Ensuring escalation processes were explicit was important to all participants. The prototypes led to queries around escalation. For example, how best to follow recommended escalation practices as set out within the chart, particularly when different combinations of circumstances may lead to more than one request for senior review in quick succession.
Instructions for use of the chart should be clear and readily available	Use of the prototypes clarified that all users would require guidance on exactly what to write in or mark within each section, and to ensure clarity on what each risk factor meant, as well as comprehensive training in escalation and response procedures.

The analysis informed a set of co-design activities with advisory group maternity professionals for the next prototype iteration.^13 41 42 46^ This included structured email exchange and online meetings facilitated by the human factors engineer or consensus facilitator to reach a professional consensus on which elements of designs 1 and 2 to incorporate in an improved draft prototype (‘Design 3’, see figure 1). Design 3 combined these elements—guided by the heuristic evaluation of step 2, the think-aloud evaluation findings of step 3 and clinical expertise—to feature:

- selective use of colour to indicate trigger values and trend lines used for recording observations,
- showing a ‘start of labour risk assessment’ on the same page as the intrapartum risk factors recorded during labour,
- an A3-sized format to improve legibility while accommodating for the content detail preferred by participants, and
- implementation of a simplified action diagram for escalation.

### Step 4: test the prototype in clinical simulations

The *evaluative* phase of co-design included clinical simulation,^13 28 41^ which is increasingly valued for its ability to support quality improvement in health systems.^60 61^ Simulations have a role in both user-centred^7 40 60 61^ and co-design approaches.^28 41^ One key advantage of clinical simulation is that rare but potentially catastrophic events or conditions can be reproduced.^62^ Design 3 (see figure 1) was tested in close approximations of real-life settings, since this is critical to safety checks, understanding how a tool might be used in practice and identifying how to improve work systems.^61^

We conducted clinical simulations involving 52 maternity professionals from five different National Health Service maternity units (online supplemental file 5). These units were recruited through convenience sampling based on availability. The simulations were designed as quality improvement activities (see Ethics approval below) guided by the ‘TEACH Sim’ framework,^63^ focusing on: (1) testing usability of the Design 3 prototype, (2) comparing care with the prototype with usual care with the unit’s existing documentation, and (3) informing the next iteration of the prototype. TEACH Sim helped to specify the simulation’s objectives, audience, scenario script, equipment, actors, and team composition.^63^

As simulation is especially well suited for conducting controlled tests exposing one group but not the other to a new prototype,^60 64^ we employed the same clinical scenario twice in each round of simulation: first with a team using usual care and the second time with a different team using the Design 3 prototype (figure 1). Facilitated by an experienced midwife from the advisory group, simulations in two units took place in situ, that is, in the participants’ own clinical settings where care is routinely performed.^60 61^ Due to clinical pressures, simulations in other units took place in a dedicated simulation laboratory or a clinical teaching setting.

Simulations were audio and video-recorded, with one camera fixed above the desk to capture participants making recordings on intrapartum tools. A trained ethnographer^65^ used a fieldnote form to record observations on aspects such as teamwork, professional roles and boundaries, communication, and social atmosphere during the simulation, with a focus on use of the intrapartum tools.

Each simulation was followed by an audio-recorded, verbatim-transcribed debrief^7 66 67^ and focus group^6 10 65^ session with the participants, facilitated by the ethnographer and an advisory group midwife using a topic guide (online supplemental file 5). The debriefing and focus group discussions with the teams involved in the simulation aided learning, through reflecting on experiences of the scenario, contextual and environment issues, safety concerns, acceptability and usability of the usual care and prototype tools, as well as identifying opportunities for better teamwork, equipment set-ups, escalation systems, and design of tools.^61 67^ The focus group discussions also helped generate further ideas to improve the draft prototype.^6 10 65^

Following the focus group, participants completed the Ottawa Acceptability of Decision Rules Instrument.^68^ This validated survey instrument further complemented assessment of reported usability,^10^ and comparison between groups providing usual care and prototype care.^68 69^

Analysis^57 70^ focused on four areas: (1) recording errors and corrections made on the prototype charts, (2) if triggers during the simulation safety checks consistently led to the required actions for safe care, (3) the role of the intrapartum tools in communication (both within the team and with those in labour and their birth partners), and (4) suggestions for improving the usability of the draft prototype. Data analysis used narrative summaries and observational checklists to code the behaviour of simulation participants based on video recordings or direct observations of the sociotechnical system during the simulations.^70^ Quantitative usability analysis assessed use errors and corrections on the charts.^19 58 71^

The findings of the simulations (table 5) informed meetings with maternity professionals from the advisory group, facilitated by the human factors engineer and consensus specialist. One key discussion point was the need to support transfer across settings, that is, from low-risk settings where intermittent auscultation is used to higher-risk settings where cardiotocography is used. The group reached a consensus on a single prototype (‘Design 4’, see figure 1). Design 4 required users to refer to a second page for actions (compared with the original single-page format—see online supplemental files 2 and 3). The group viewed this as an acceptable trade-off given that the single prototype would support transfer across settings.

**Table 5:** Examples and key findings of the analysis of the simulations

Example of data analysis	Key finding
**Errors and corrections on charts** Completed charts were evaluated against the scenarios and video recordings to identify errors and corrections when using the prototype chart during the simulations	Across prototype care at the five sites: 13 errors in recorded observations 7 in fetal or maternal heart rate recordings, with likely minimal or no impact on safety of care 6 in action diagram actions, with potentially significant impact on safety of care 10 corrections, defined as cases where the original incorrect mark/value was changed to the correct value 5 in fetal or maternal heart recordings 4 in action diagram actions 1 in gestation period
**Safety of prototype care vs usual care** Video analysis of triggers during the simulation (eg, vaginal bleeding, pathological cardiotocography) leading to the required actions for safe care	When using the draft prototypes, triggers during the simulation consistently led to the required actions for safe care as prescribed in the prototype action diagram (eg, transfer to obstetric-led centre, expediting birth). These actions were generally also undertaken in the usual care simulations.
**Reference to prototype chart during team member exchanges** Video analysis of verbal and visual reference to the prototype chart in identified exchanges between team members, and qualitative analysis of focus group discussions and ethnographic notes	The prototype chart was verbally and visually referenced in 84% of 50 identified exchanges between team members during high-risk scenarios, but was referenced in only 33% of 18 identified exchanges during low-risk scenarios. The focus groups suggested this may have been a function of the relative simplicity of the low-risk scenarios, which required relatively few events to recall and explain during the team member exchange. Ethnographers and participants noted that referring to prototype chart enabled more rapid transfer of information and understanding of the clinical situation: ‘The obstetrician actually got the picture of this woman very quickly, as to what was happening because she hadn’t been in the room at all during the first part of the simulation, so she was coming into the room as she possibly might be in a real-life situation.’
**Team communication and decision-making** Qualitative analysis of focus group discussions, focusing on usability of the prototype for improving team communication and decision-making	Ethnographers and participants noted that the visual flow of recorded observations and associated triggers in the action diagram improved team communication and decision-making: ‘I did find it was easier to escalate. […] It was more of an agreed decision there, like we were all in agreement with what the plan was, rather than just being like, different doctors make different plans.’
**Communication with those in labour and their partners** Qualitative analysis of ethnographic notes and focus group discussions, focusing on quality and quantity of communication with service user actors	The ethnographic observations indicated tendencies for midwives to focus more on the paperwork than on communicating with those in labour—both in the usual care setting as well as when using the prototype tools. Participants suggested in the focus groups that the simulation situation contributed to this, and that the effect would diminish with familiarity and training with the chart, but also that enhanced chart design might further encourage optimal communication with those in labour.
**Suggested areas of improvement** Qualitative analysis of focus group discussions, synthesising suggestions of participants for improving the prototype	One potential area of improvement was the integration of the separate draft prototypes (one for low-risk settings with use of intermittent auscultation, and one for high-risk settings with use of cardiotocography) into a single prototype. Perceived advantages were facilitation of tracking of risk factors across settings, and reduction of error risk when transcribing from one chart to the other: ‘Actually, we do look after people who start off on intermittent auscultation and then rupture the membranes with meconium and then have to go on a CTG [cardiotocography].’ ‘I like that idea, having them both on the same piece of paper but just really, really clearly demarked, this is the intermittent auscultation.’

### Step 5: generating a final prototype using co-design workshops

Step 4 identified a need for further input to (i) improve use of the prototype in terms of communication with the person in labour and (ii) finalise the action diagram. To complete the *evaluative* co-design phase,^28^ this was addressed with the advisory group through workshops. These have shown potential for practical and effective ways to finalise a prototype.^13 17 18 39 41 46^ To address potential power imbalances, workshops were organised with subgroups (figure 1). Facilitators supported agenda-setting, procedures, and consensus rules,^72 73^ and were mindful of power dynamics.^44^

The PPI facilitators introduced Design 4 to the five service user representatives and gained feedback on it during three discussion workshops (figure 1), exploring in particular how the prototype might impact communication with those in labour. The maternity professionals and human factors engineer joined the discussions upon invitation by the PPI facilitators or service users. These workshops led to the inclusion of an additional item—‘is the woman concerned?’—in the final prototype, as this was a key proposal made by the representatives.

To address the identified use difficulties with the flow chart actions, an alternative grid format (vs the original flow chart format) was developed.^10 46^ The human factors engineer facilitated three workshops with maternity professionals from the advisory group, in which the flow chart and grid formats were used alongside each other with reference to written clinical scenarios (figure 1).^10^ They reached a consensus that the grid layout provided better usability—through its better conveyance of the data^34 54^—and should be implemented in the final prototype. The final prototype (see online supplemental file 6) was prepared by the human factors engineer and graphic designer, reviewed by the advisory group and considered ready for use in large-scale testing.

## Discussion

Clinical practice tools have not routinely benefited from systematic combination of user-centred design methods and co-design principles applied to their development,^6 17 18^ despite the availability of well-established techniques with a good track record in improving design and usability in a range of clinical applications.^11 13 16 29 30^ One likely reason for this is the limited practical guidance about how to deploy these approaches in a pragmatic yet systematic manner for development of clinical practice tools. The framework (FRESCO) proposed in this article codifies existing user-centred design methods and co-design principles into practical guidance for enabling mobilisation of multiple forms of expertise for development of clinical practice tools. Our case study illustrates application of the framework in an area of pressing need, leading to a viable track-and-trigger prototype tool ready for large-scale testing. The study also helps to address the call for better reporting of healthcare improvement activities that align with principles of co-design.^18 39 74^

FRESCO builds on an established co-design framework (table 1),^28–30^ including use of pre-design, generative, and evaluative phases that can inform future post-design implementation phases with the produced prototype. One of its contributions is in sensitising developers of clinical practice tools to systematic consideration of the needs and priorities of users—through application of principles of collective creativity and inclusivity central to co-design into a series of actionable steps^25–27^—while employing a user-centred design approach that supports safety, effectiveness, and efficiency.^22–24^ The case study also illustrates that employment of FRESCO is consistent with a design process moving from medium to high *structural restrictiveness*.^55^ The generative phase started with various concept prototypes that encouraged the co-design group to explore alternative ideas, which helped prevent the risk of premature closure around one solution. During the evaluative phase, the best elements of the concept prototypes were then integrated through iterative cycles into a single prototype, using high structural restrictiveness to increase decision-making precision.^55^

Findings of the case study suggest that FRESCO supports inclusive ways of co-designing prototype clinical practice tools and enabled improvements based on voices that are often under-represented in development of clinical practice tools. As an example, a novel prompt—‘is the woman concerned?’—was included in the prototype to help ensure optimal communication with those in labour and their birth partners, following input of service user representatives. This helped address the imperative to include patient/family concern in track-and-trigger systems^75^ as well as the broader concern to listen better to families and involve them in their own care.^76^ The in situ simulations helped to understand how the prompt could be best used in practice. Key to achieving co-design in this way is commitment to inclusion, facilitation that focuses on hearing everyone’s voices and managing power dynamics through, for example, organising separate activities for service users when needed. These findings suggest that FRESCO can contribute to the need for effective ways of co-design with patients, as called for in models for co-creation of healthcare services.^77^

### Strengths and limitations of the framework

While FRESCO helped develop a prototype track-and-trigger tool, further evaluation will be needed to determine clinical and service user experience, efficiency, implementability, sustainability of change, impact on clinical outcomes, and any unintended consequences.^78 79^ Piloting and large-scale, national testing will be important in supporting this. Further examples of use cases outside of this context would help to refine and test the framework, for example to: (1) determine whether clinical practice tools produced using the framework offer advantages over others, (2) establish the resourcing needed for minimal and optimal execution of each step, and (3) assess the extent to which steps may need to be adapted for use in lower-resourced settings. There is also a need to generate learning on how to sustain engagement and involvement of users in the design process.

Although the resource implications of using FRESCO are significant, so too are the costs of developing the technical components of clinical practice tools.^14^ Moreover, deploying suboptimally designed tools introduces multiple risks and potential for waste.^3 5 12 71^ Ultimately, FRESCO could help to prevent the characteristic dysfunctions associated with exclusively bottom-up or top-down innovation for quality improvement,^80^ such as lack of access to specific expertise common in locally led, bottom-up approaches,^15^ and risk of perverse incentives associated with top-down approaches.^81^ For example, using the framework as a practical guide to developing a prototype clinical practice tool could help prevent suboptimal implementation owing to inadequate or absent exploration of usability or acceptability,^7 38 78 82 83^ or waiting until the end of the development cycle when the sunk costs may limit improvement.^7 12 83^

### Limitations of the case study

The pandemic conditions in which the case study was conducted imposed some limitations, including the need to adapt established in-person think-aloud methods and conduct of observations. These adaptations did highlight the flexibility inherent to our proposed framework. Ongoing pressures caused by the pandemic also required the use of convenience sampling of units for the simulations and use of clinical simulation laboratories instead of in situ settings in some units, so representativeness was difficult to determine.

## Conclusion

The proposed framework (FRESCO), combining user-centred design methods and co-design principles, was successfully deployed to develop a prototype clinical practice tool for detecting and responding to possible fetal deterioration during labour. By codifying existing methods and principles into a single actionable framework, FRESCO has potential to facilitate pragmatic, flexible, and inclusive co-design of clinical practice tools using methods that can be standardised, replicated, and potentially scaled when needed, but will require further evaluation. Future work can also help identify the kinds of applications the framework works best for and where its limits lie.

## Footnotes

https://thiscovery.org/about
