# Supplementary material for: How to co-design a prototype of a clinical practice tool: a framework with practical guidance and a case study
Source: BMJ Qual Saf. 2023 Dec 12;33(4):258–70. doi: 10.1136/bmjqs-2023-016196 (PMC10982632; doi:10.1136/bmjqs-2023-016196)
Supplement: Supplementary data [file bmjqs-2023-016196supp001.pdf]

How to co-design a prototype of a clinical practice tool: a framework with practical guidance and a case study

Supplements

Supplement 1: page 1

Supplement 2: page 2

Supplement 3: page 8

Supplement 4: page 11

Supplement 5: page 12

Supplement 6: page 14

References: page 17

Supplement 1

Advisory group members (n=22) involved in co-design of the prototype chart.

Maternity professionals (n=12)	Those with lived experience of using maternity service (n=5)	Other specialists (n=5)
• Midwives experienced in hospital and/or community birth settings (n=4)<br>• Midwife with expertise in maternity education<br>• Consultant midwife<br>• Trainee obstetrician<br>• Consultant obstetricians (n=5)	• Service users with a range of maternity experiences and experience of advocating for improvement and inclusion of under-represented voices (n=5)	• Human factors engineer<br>• Graphic designer<br>• Consensus-building specialist<br>• PPI facilitation specialists (n=2)

Woodward M, *et al. BMJ Qual Saf* 2024; 33:258–270. doi: 10.1136/bmjqs-2023-016196

Supplement 2

*The examples below are "blurred" versions of some of the prototypes used across the co-design process, i.e. the first set of prototypes developed as part of **Step 2** and the final prototype agreed on during **Step 5**. "Non-blurred" prototypes cannot be presented due to reasons related to confidentiality.*

Woodward M, *et al. BMJ Qual Saf* 2024; 33:258–270. doi: 10.1136/bmjqs-2023-016196

Example: Design 1 for IA, to be printed on A4

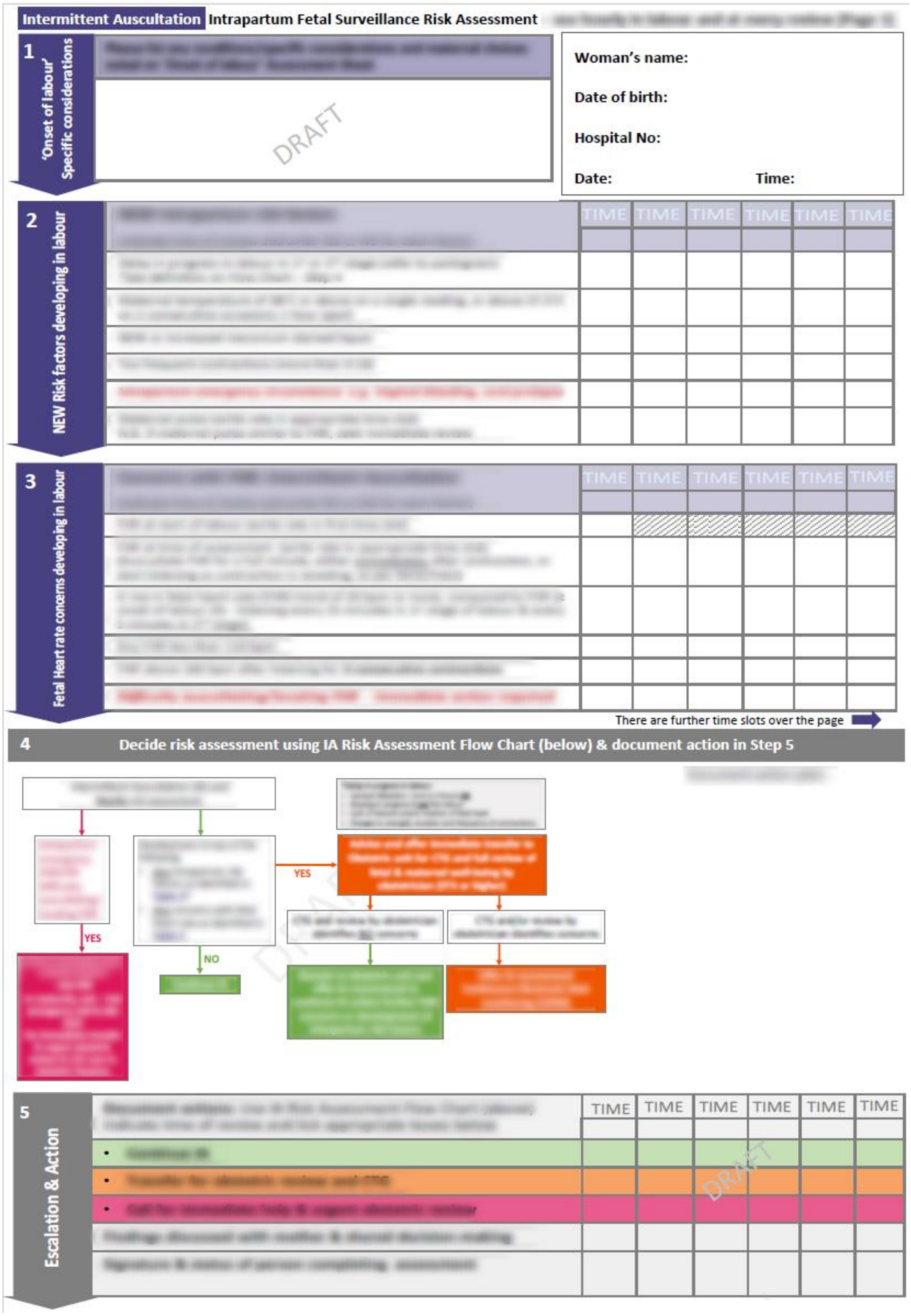

Example: Design 1 for CTG, to be printed on A4

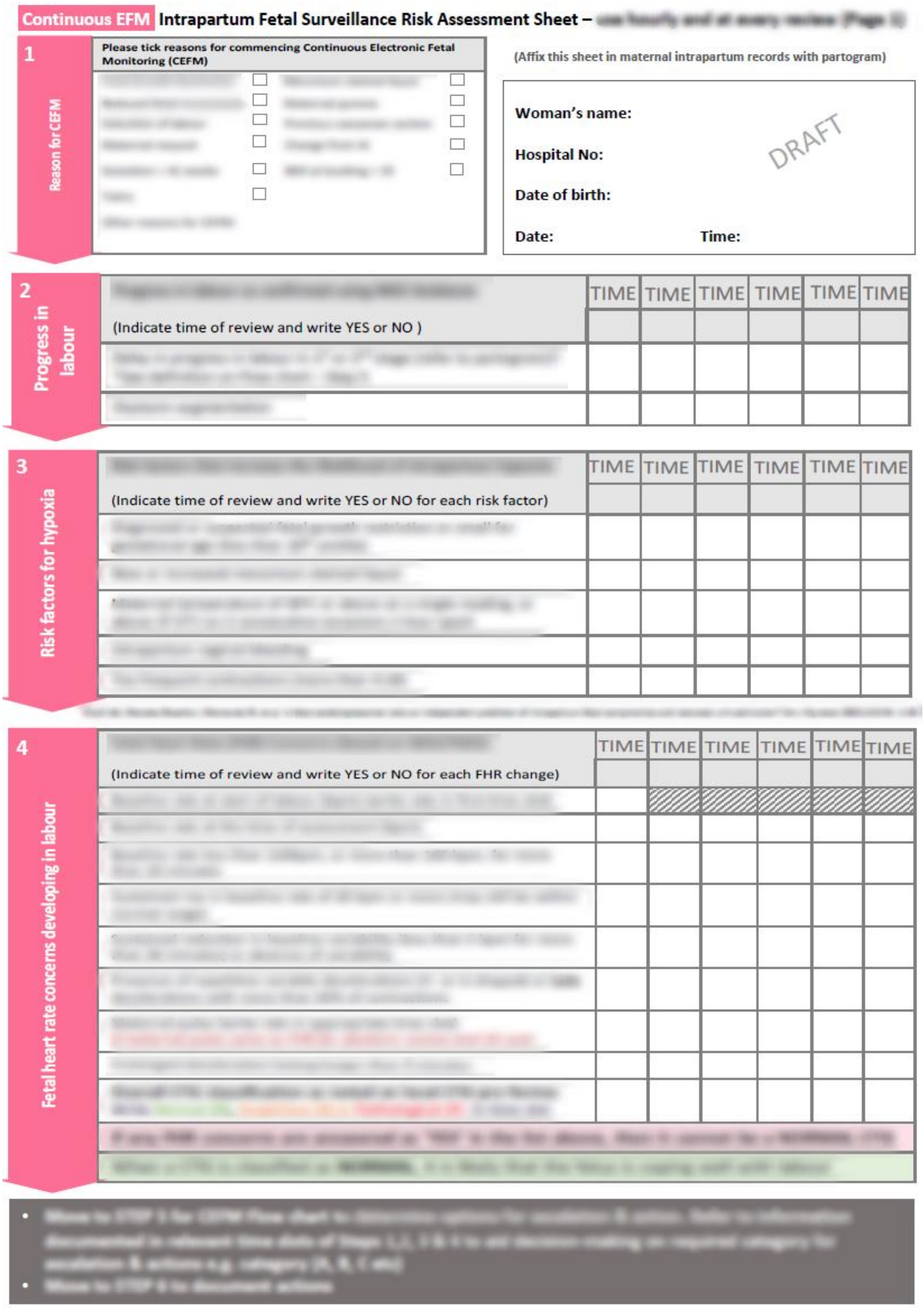

Continuous EFM **Intrapartum Fetal Surveillance Risk Assessment Sheet –** [illegible]

1 Reason for CEFM

Please tick reasons for commencing Continuous Electronic Fetal Monitoring (CEFM)

[illegible]

(Affix this sheet in maternal intrapartum records with partogram)

Woman's name:

Hospital No:

DRAFT

Date of birth:

Date: **Time:**

2 Progress in labour

[illegible] (Indicate time of review and write YES or NO)	TIME	TIME	TIME	TIME	TIME	TIME
[illegible]						
[illegible]						

3 Risk factors for hypoxia

[illegible] (Indicate time of review and write YES or NO for each risk factor)	TIME	TIME	TIME	TIME	TIME	TIME
[illegible]						
[illegible]						
[illegible]						
[illegible]						
[illegible]						

[illegible]

4 Fetal heart rate concerns developing in labour

[illegible] (Indicate time of review and write YES or NO for each FHR change)	TIME	TIME	TIME	TIME	TIME	TIME
[illegible]						
[illegible]						
[illegible]						
[illegible]						
[illegible]						
[illegible]						
[illegible]						
[illegible]						
[illegible]						

[illegible]

[illegible]

- [illegible]
- [illegible]

4

Continued from previous page (rear side of A4 printed version)

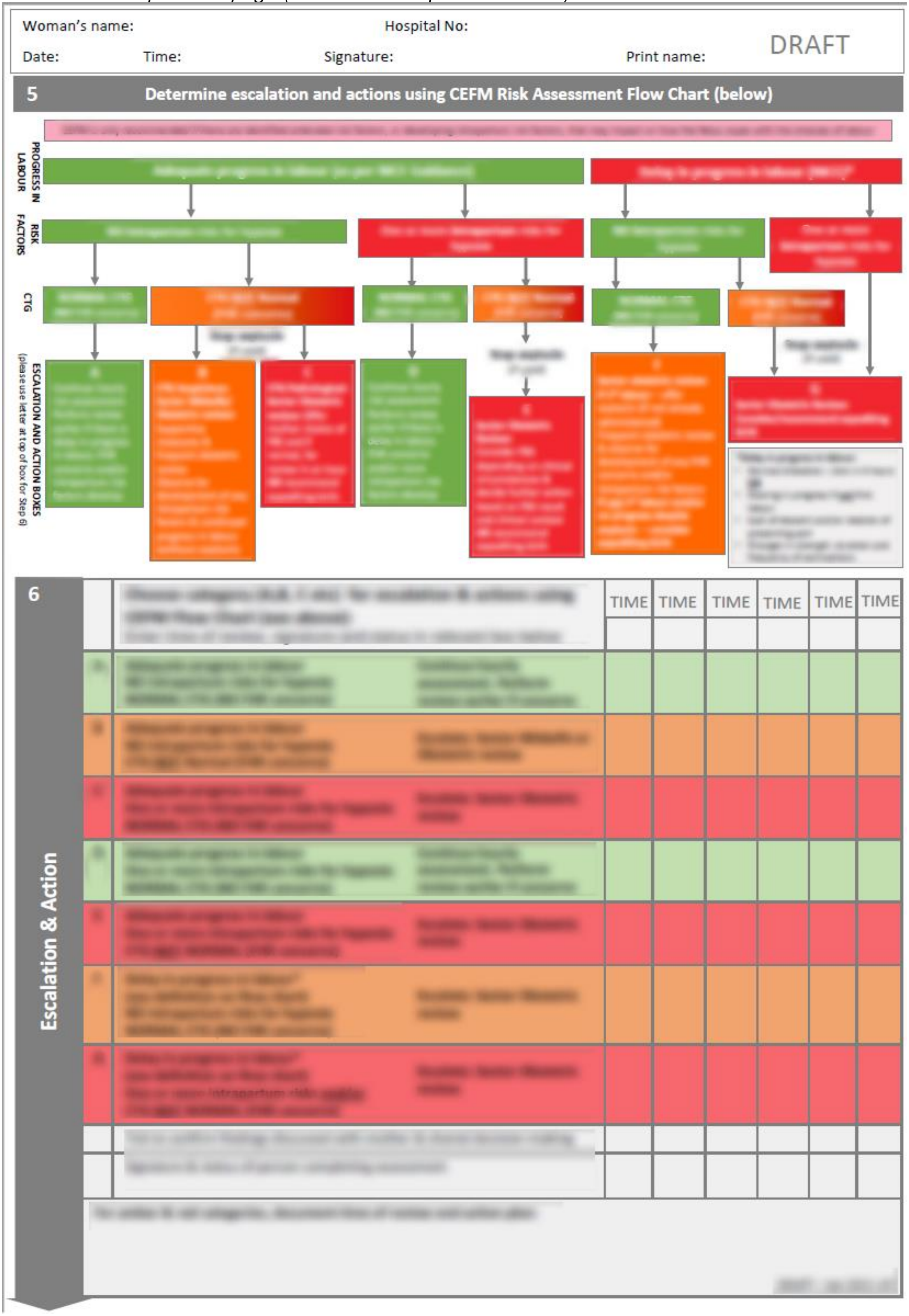
Woman's name: Hospital No:
Date: Time: Signature: Print name: DRAFT

5 Determine escalation and actions using CEFM Risk Assessment Flow Chart (below)

PROGRESS IN LABOUR
RISK FACTORS
CTG
ESCALATION AND ACTION BOXES (please use letter at top of box for Step 6)

6 Escalation & Action

TIME TIME TIME TIME TIME TIME

5

Example: Design 2 for IA, to be printed on A4

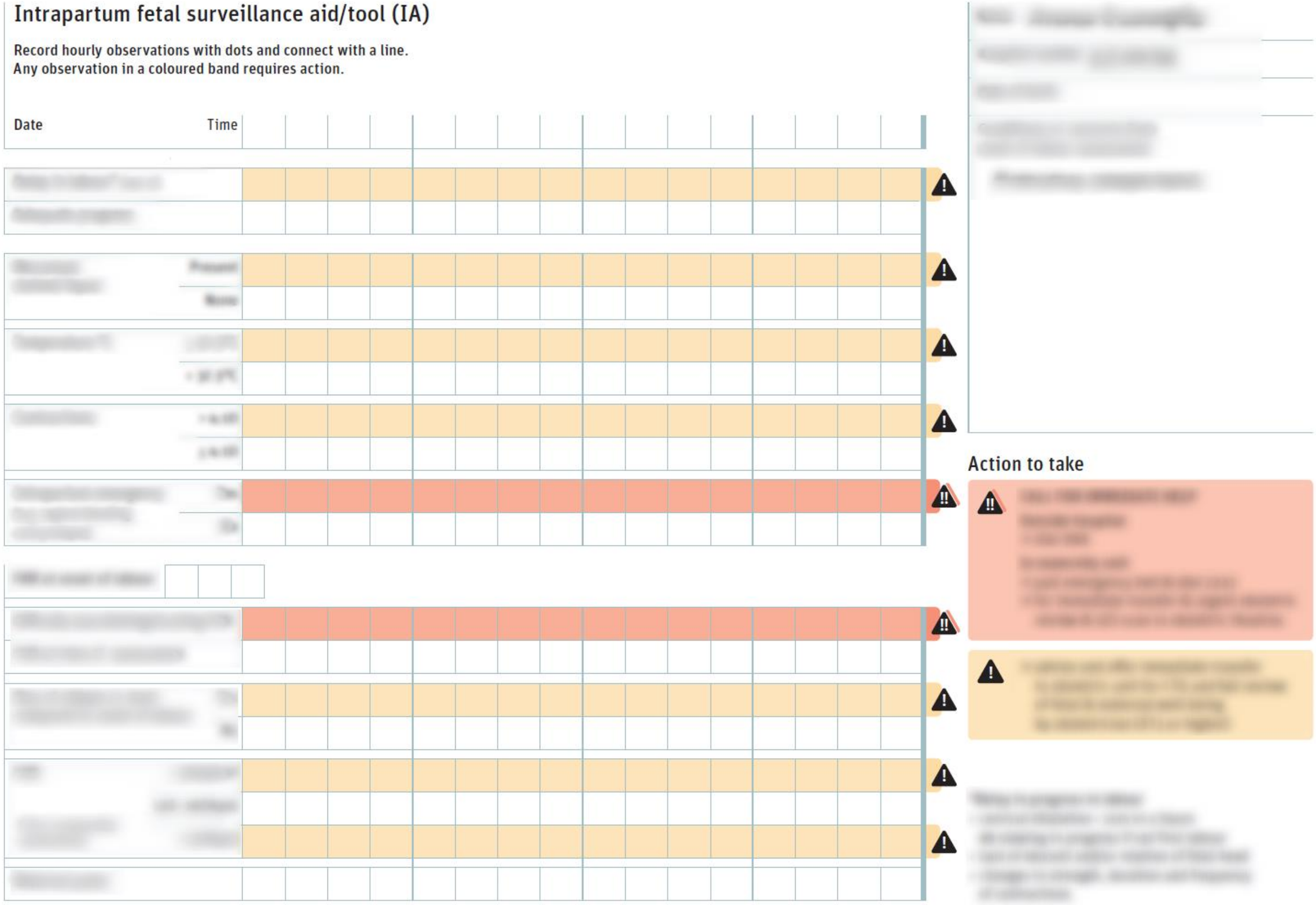

Example: Design 2 for CTG, to be printed on A4

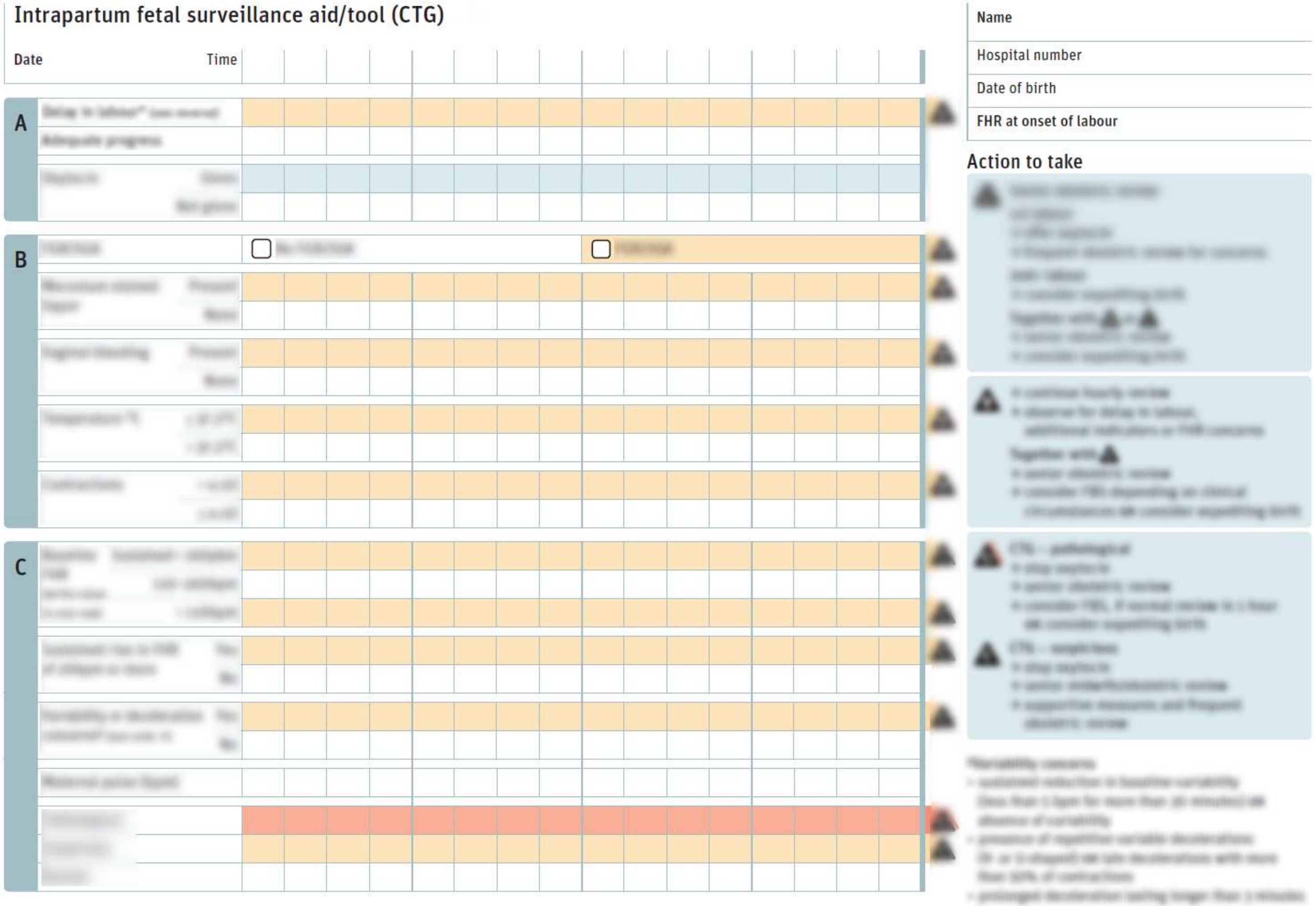

Supplement 3

Key components of context of use for the chart, as established in a scoping exercise regarding electronic fetal monitoring in maternity units in the United Kingdom (UK).

Component	Explanation
Holistic	Equal prominence to fetal heart rate features and other intrapartum risk factors
Use in all settings	Appropriate for use in all maternity settings in the UK, including fetal heart rate monitoring using intermittent auscultation and cardiotocography in obstetric-led settings, midwife-led “alongside” hospital settings, and community settings
Accommodate for different CTG classification systems	Allowing use with the various fetal heart rate feature classification systems used across units in the UK
Easy to use	Straightforward and quick to complete for midwives during labour, and straightforward and quick to review for obstetricians, including during high and low-light settings of day and night shifts
Complementary with other documentation	Amenable for integration with other required intrapartum documentation, such as the partogram that is used in most birth settings and units in the UK[1]
Paper-based prototype	Units across the UK currently vary in their use of paper or online intrapartum documentation, with the most common context being the use of paper-based charts for documenting vital signs.[2] This, together with human factors engineering guidance that recommend prototyping paper versions before online versions,[3,4] led to a focus on a paper-based prototype that could inform a digital version at a later stage.

Examples of design features applied to Design 2 when compared to Design 1 (see Figure 1), linked to user-interface design principles originating from the field of human factors engineering[5-9]

Design feature	Detail	User-interface design principles applied[5-9]
**Layout**		
Action section	Action section is placed adjacent to observations.	Minimises load on working memory and potential for error when cross-referencing observation to action.
Single box for recording time	The user is only required to write the time of observations once.	Simplifies design and reduces the time to complete.
Column design	Thicker vertical lines are drawn every four columns.	Supports user to track down a set of observations without ‘column shift’.

Number of columns	16 columns (time slots) are included per sheet.	Better match of task requirements. NICE guidance states that the average first labour is 8 hours and unlikely to exceed 18 hours.
Overall layout	Observations section positioned on the left, action section on the right, woman's details are top-right.	Top-left is the prime position for attention and is thus used for the primary and frequent task of recording observations.<br>Natural progression from left to right to translate observations to action.<br>Top-right is commonly used on NHS forms to record patient details and thus follows convention.
**Notation, colour, font**		
Notation for recording observations	Dots and joining lines are used to record observations.<br>A series of normal observations are drawn as a straight line, with variation drawn as an ascending or descending line.	Anomalies are easier and quicker to detect when there is a break from a whole figure; this is based on the Gestalt theory of perception.<br>In the case of the tool, a deviation from a straight line may indicate a "trigger".
Colour	Orange and red are used for out-of-range observations ("triggers").<br>Saturation of red is higher than that of orange.	Supports user population expectations as orange and red follow the convention used on early warning systems.<br>Distinguishing colours by saturation, brightness and hue enhances perceived difference and retains distinction in greyscale prints.
Visual coding	Colours and symbols are used to match a trigger to an action.<br>Both colour and symbol used to communicate meaning.	Use of the same colour to match an observation with the corresponding action.<br>A triangle symbol was also used to indicate a trigger. A second (redundant) code is useful in case the primary code is not available (for example with grayscale prints).
Typeface	Sans-serif typeface selected, with a font size between 9-11 points	The "Unit Rounded Pro" is a clearly legible font. Font size follows recommendations for printed text.
Consistent use of colour, capitalisation, typeface	Consistent typeface, text justification and use of capitalisation.<br>Colour codes retain the same meaning wherever used.	Consistent design features are quicker and less effortful to interpret.
Terminology	The terms used are familiar to users. Abbreviations are avoided and users were consulted on suitability of acronyms.	Facilitating comprehension.

Woodward M, *et al. BMJ Qual Saf* 2024; 33:258–270. doi: 10.1136/bmjqs-2023-016196

Examples of alternative design elements of "Design 1" and "Design 2" of the draft prototype charts.

	Design 1	Design 2
**Page size and format**	A4 portrait	A4 landscape
**How to record observations**	YES or NO recorded in a table	Dots and lines marked in colour-coded rows
**Number of timeslot columns for consecutive (hourly) recordings**	6 on a single chart	16 on a single chart
**Link between recordings and actions**	Flowchart diagram with actions differentiated based on "YES" in observations table	Actions described in boxes adjacent to related colour-coded rows
**Detail on fetal heart rate features**	Separate rows with details for each fetal heart rate concern	Rows combining several related fetal heart rate concerns
**Inclusion of "start of labour risk assessment"**	Yes	No (assumed to be presented in separate antenatal documentation)

Woodward M, *et al. BMJ Qual Saf* 2024; 33:258–270. doi: 10.1136/bmjqs-2023-016196

Supplement 4

Characteristics of the participants in the think-aloud formative evaluations, including nine midwives and six obstetricians working across the full range of maternity settings within England.

Role	Unit type	Number of participants
**Midwives**		
Band 8-9	Obstetric only	1
Band 5-7	Community	1
	Community and freestanding midwifery unit	1
	Community and obstetrics	1
	Obstetric and alongside midwifery unit	2
	Obstetric only	3
**Obstetricians**		
Trainee	Obstetric and alongside midwifery unit	1
	Obstetric only	3
Consultant	Alongside midwifery unit	1
	Obstetric only	1

Semi-structured interview guide used following the think-aloud exercises with Design 1 and 2

1) **Thinking about the two versions of the tool you have completed:**
   a) What is your view on recording the response action on the tool versus elsewhere?
   b) Which elements would you take forward from each design to the next design iteration?
   c) What is your view on including: a record of individual fetal heart rate features on the form versus an overall categorisation (normal/suspicious/pathological) and fetal heart rate?
   d) Which was your preferred version, and why?
   e) What changes might improve the design of your preferred version?
   f) Which elements caused confusion or difficulties?

2) **Thinking about what it might be like to use the tools in practice:**
   a) Would this tool fit with existing documentation systems on your unit?
   b) How might this tool help or hinder escalation?

Supplement 5

Characteristics of units* where simulations testing took place.

	Site 1	Site 2	Site 3	Site 4	Site 5
**Type of hospital**	District general hospital	Tertiary	Tertiary	Tertiary	District general hospital
**Region**	South West	East Midlands	South West	Greater London	South East
**Birth setting services**	Obstetric-led<br>Alongside<br>Freestanding<br>Home births	Obstetric-led<br>Alongside<br>Freestanding<br>Home births	Obstetric-led<br>Alongside<br>Freestanding<br>Home births	Obstetric-led<br>Alongside	Obstetric-led<br>Alongside
**Paper or digital tools in usual care**	Paper	Paper	Paper	Digital, with paper partogram in low-risk labour	Paper
**Electronic fetal monitoring guidelines**	NICE	FIGO (plus physiological approach)	FIGO	NICE	NICE (plus physiological approach)

** Due to the COVID-19 pandemic, participating units were selected primarily on their ability to facilitate simulation sessions and on their availability, but did represent diversity of maternity settings*

Number and professional backgrounds of the 61 participants in the simulations.

	Site 1	Site 2	Site 3	Site 4	Site 5
**Midwives (n)**	10	8	10	10	9
**Trainee obstetricians (n)**	2	1	0	0	2
**Consultant obstetricians (n)**	2	3	2	2	0
**Total (N)**	14	12	12	12	11

Examples of topic guide questions used in the post-simulation focus group

Did you encounter any difficulties or confusion in completing the tool? If so please explain *(prompts: workflow, terminology, legibility, layout; finding you weren't using it)*
Was it clear what action to take when the condition of the woman/baby started to deteriorate?
Compared to usual practice, what effect did the tool have on communicating with your colleagues about what was happening? *(Probe for differences in communication midwife-midwife and midwife-doctor as appropriate).*
What changes might improve the design or content of the tool? *(prompts: workflow, terminology, legibility, layout). In an ideal world?*
The risk factors list at the side of the tool is based on previous robust research on clinical indicators. What do you think about a) having these on the tool b) the position of this list on the tool; c) the order in which the factors are set out?

Woodward M, *et al. BMJ Qual Saf* 2024; 33:258–270. doi: 10.1136/bmjqs-2023-016196

What aspects of the tool you have just used worked well?
Was the amount of time it took to complete the tool acceptable or too long?
What effect (if any) did the tool have on your communication with the woman and her partner?
Overall, did the tool support you or hinder you in providing care to the woman and her baby?
What do you think your colleagues would say about the tool? (Is that different for midwives from obstetricians?) *[useful for understanding wider context and as 'othering' technique to elicit concerns that participants may feel wary about owning]*
What effect did the paperwork have on your communication with your colleague/s and on communication with the woman/birth partner? (positive/neutral/negative?)
How useful was the risk factors list at the side of the tool? When did you refer to it? For example, just at the beginning of the sim or more frequently, e.g. prior to escalation
Thinking about what it might be like to use the tool in practice:<br>• How well would this tool fit with existing documentation systems on your unit?<br>• How might this tool help or hinder escalation?<br>• One idea is to combine both IA and CTG fetal heart rate monitoring into a single tool. What do you think are the benefits and drawbacks of combining the two?<br>• Another idea we are exploring is combining the tool with the partogram. What do you think are the benefits and drawbacks of that?

Supplement 6

*The examples below are "blurred" versions of some of the prototypes used across the co-design process, i.e. the first set of prototypes developed as part of **Step 2** and the final prototype agreed on during **Step 5**. "Non-blurred" prototypes cannot be presented due to reasons related to confidentiality.*

Woodward M, *et al. BMJ Qual Saf* 2024; 33:258–270. doi: 10.1136/bmjqs-2023-016196

Final prototype, to be printed on A3

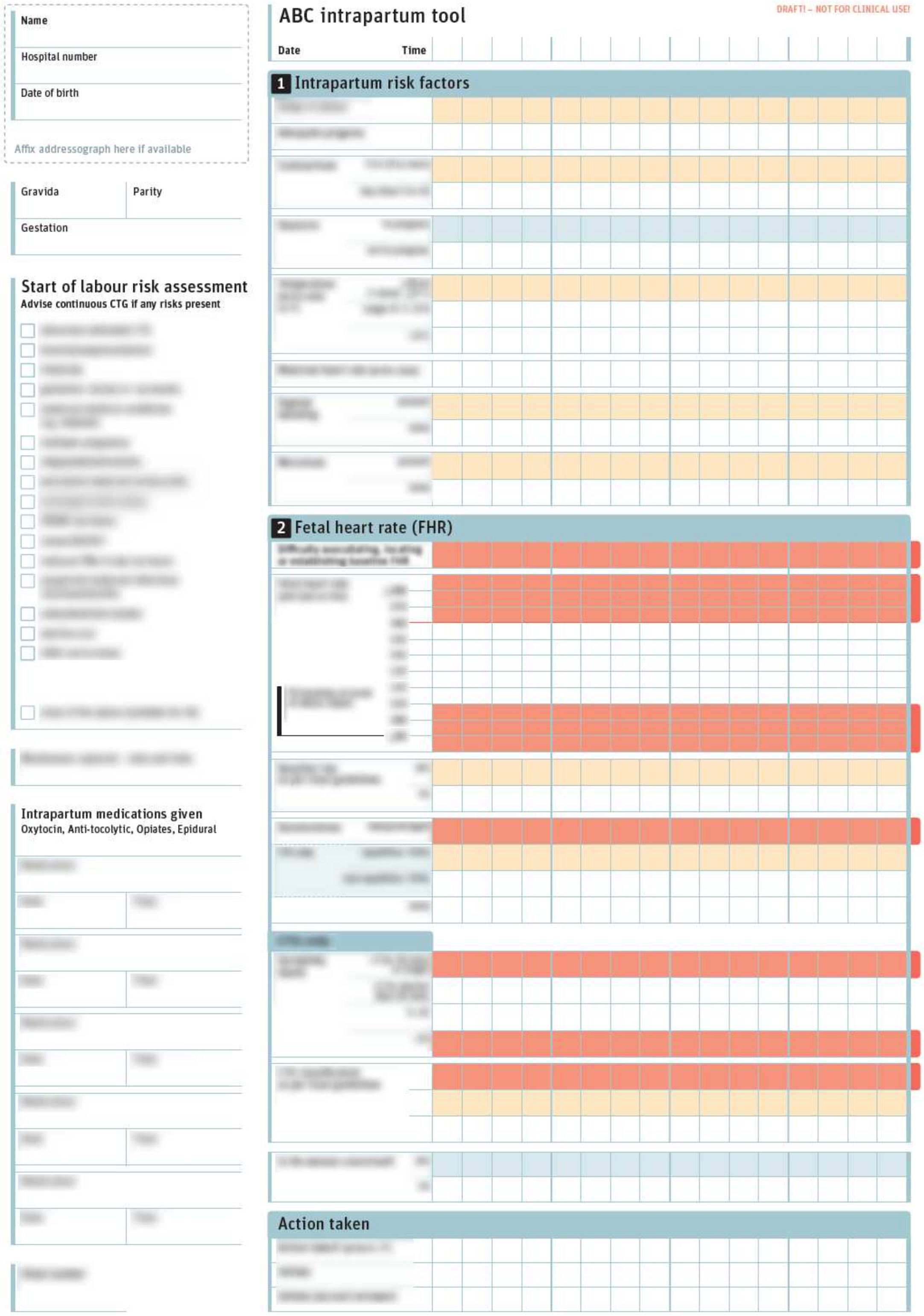

Continued from previous page (rear side of A4 printed version)

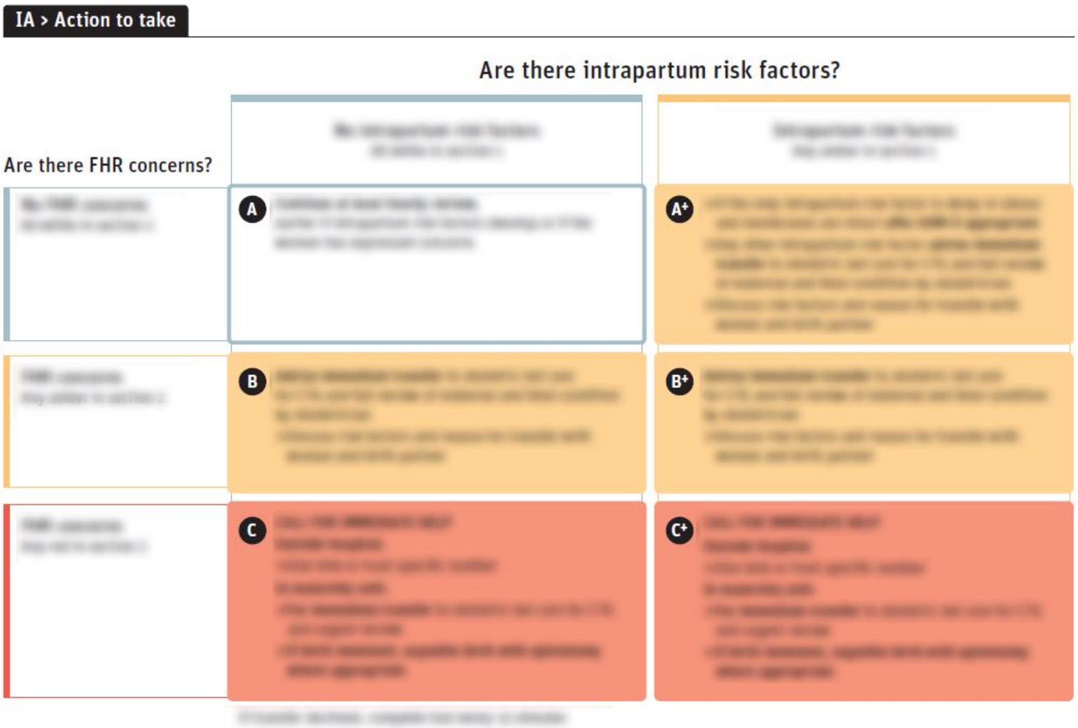

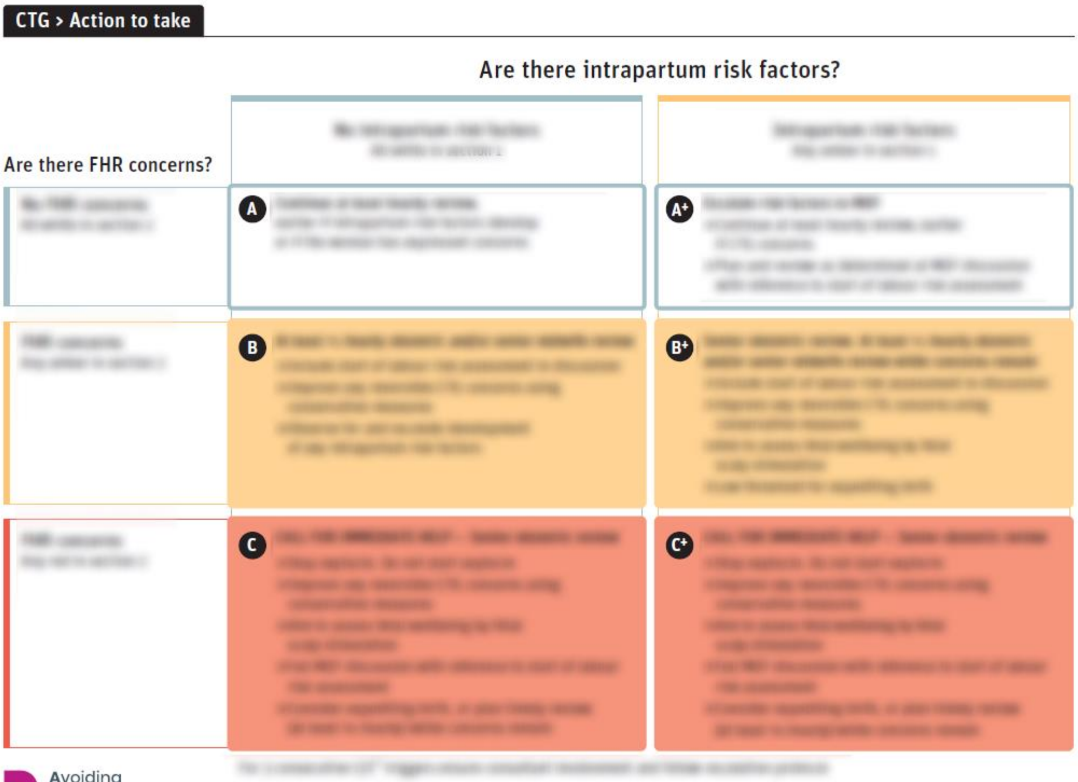

Avoiding Brain Injury in Childbirth

References

1. Lavender T, Tsekiri E, Baker L. Recording labour: a national survey of partogram use. *British Journal of Midwifery* 2008;16(6):359-62. doi: 10.12968/bjom.2008.16.6.29593
2. Isaacs R, Smith G, Gale-Andrews L, et al. Design errors in vital sign charts used in consultant-led maternity units in the United Kingdom. *International Journal of Obstetric Anesthesia* 2019;39:60-67. doi: 10.1016/j.ijoa.2019.01.001
3. Camburn B, Viswanathan V, Linsey J, et al. Design prototyping methods: state of the art in strategies, techniques, and guidelines. *Design Science* 2017;3 doi: 10.1017/dsj.2017.10
4. Sefelin R, Tscheligi M, Giller V. Paper prototyping - what is it good for? a comparison of paper- and computer-based low-fidelity prototyping. CHI '03 Extended Abstracts on Human Factors in Computing Systems. Ft. Lauderdale, Florida, USA: Association for Computing Machinery, 2003:778–79.
5. Dashevsky SG. Check-reading accuracy as a function of pointer alignment, patterning, and viewing angle. *Journal of Applied Psychology* 1964;48(6):344-47. doi: 10.1037/h0042066
6. Sanders MSM, E. J. Human Factors in Engineering and Design. 7th ed: McGraw-Hill 1993.
7. Shneiderman B, Plaisant C, Cohen M, et al. Designing the User Interface: Strategies for Effective Human-Computer Interaction, Global Edition. Harlow, UNITED KINGDOM: Pearson Education, Limited 2017.
8. Preece MH, Hill A, Horswill MS, et al. Applying heuristic evaluation to observation chart design to improve the detection of patient deterioration. *Appl Ergon* 2013;44(4):544-56. doi: 10.1016/j.apergo.2012.11.003 [published Online First: 20121208]
9. Zhang J, Johnson TR, Patel VL, et al. Using usability heuristics to evaluate patient safety of medical devices. *Journal of Biomedical Informatics* 2003;36(1):23-30. doi: https://doi.org/10.1016/S1532-0464(03)00060-1

Woodward M, *et al. BMJ Qual Saf* 2024; 33:258–270. doi: 10.1136/bmjqs-2023-016196
